# Integration of omics sciences to advance biology and medicine

**DOI:** 10.1186/1559-0275-11-45

**Published:** 2014-12-15

**Authors:** Emily S Boja, Christopher R Kinsinger, Henry Rodriguez, Pothur Srinivas, on behalf of Omics Integration Workshop Participants

**Affiliations:** Office of Cancer Clinical Proteomics Research, National Cancer Institute, National Institutes of Health, Bethesda, MD USA; Division of Cardiovascular Sciences, National Heart, Lung and Blood Institute, Bethesda, MD USA

**Keywords:** Omics integration, Omics science, Clinical application, Risk prediction, Proteomics, Metabolomics, Genomics

## Abstract

In the past two decades, our ability to study cellular and molecular systems has been transformed through the development of omics sciences. While unlimited potential lies within massive omics datasets, the success of omics sciences to further our understanding of human disease and/or translating these findings to clinical utility remains elusive due to a number of factors. A significant limiting factor is the integration of different omics datasets (i.e., integromics) for extraction of biological and clinical insights. To this end, the National Cancer Institute (NCI) and the National Heart, Lung and Blood Institute (NHLBI) organized a joint workshop in June 2012 with the focus on integration issues related to multi-omics technologies that needed to be resolved in order to realize the full utility of integrating omics datasets by providing a glimpse into the disease as an integrated “system”. The overarching goals were to (1) identify challenges and roadblocks in omics integration, and (2) facilitate the full maturation of ‘integromics’ in biology and medicine. Participants reached a consensus on the most significant barriers for integrating omics sciences and provided recommendations on viable approaches to overcome each of these barriers within the areas of technology, bioinformatics and clinical medicine.

## Introduction

The past two decades have been witness to an explosion of data stemming from the development and gradual maturation of ‘omics’ technologies and bioinformatics. Today, whole-genome sequencing has become a routine research tool, and state-of-the-art proteomic technologies have caught up to genomics in the past few years in terms of coverage as evidenced by their ability to identify a large percentage of all observed human gene products, including functionally significant alternative splice variants [1–4]. Nevertheless, the omics mindset has not yet permeated the broad biological and clinical community. Of the ~20,000 genes in the human genome, only 10% have 5 or more publications [5], while one gene, p53 that regulates the cell cycle and functions as a tumor suppressor, is the subject of over 56,000 articles in scientific literature. Clearly, our technological abilities to generate large amounts of data from molecular systems have advanced enormously, but the ability to translate this information for use in the clinic remains elusive due to a number of factors. One key reason postulated is that while individual omics domains yield distinct and important information, no single omics science is sufficient to facilitate a comprehensive understanding of the complex human biology and physiology. Additionally, there are logical scientific steps missing in leaping from a lack of information on 90% of the proteins to clinical use. The integration of omics sciences bioinformatically remains a challenge and thus a limiting factor in fully extracting biological meaning from the mounds of data being generated. For instance, the NCI’s The Cancer Genome Atlas (TCGA) integrated multiple data types to identify three mutually exclusive pathways that affect the development of glioblastoma multiforme (e.g., RTK, TP53, RB) [6], suggesting that the presence of one aberration removes the selective pressure for a second aberration. This example demonstrates the immediate value of data integration since these pathways were not observed from data in isolation (either from mutations, copy number changes, or other measurements). Omics integration is the next logical and necessary step in propelling systems biology and medicine forward and potentially allowing for its use in the clinic. NCI’s Clinical Proteomic Tumor Analysis Consortium (CPTAC) is one such multi-institutional initiative that employs proteogenomic integration to better enhance our understanding of cancer biology using genomically characterized tumors [7], and there are similar international efforts such as uniting the chromosome-centric human proteome project with the Encyclopedia of DNA Elements (ENCODE) [8].

## Executive summary

In light of previous workshops addressing the challenges and opportunities of clinical proteomics in biology and medicine [9, 10] and the advancement of proteogenomic science, the NCI and NHLBI organized a workshop focusing the topic of integrating omics datasets obtained from multi-omics technologies to provide broader insights into disease pathophysiology. The workshop was held on the National Institute of Health (NIH) campus in Bethesda, MD on June 19 and 20, 2012 with participants from a diverse variety of scientific expertise. Herein, this report summarizes the major challenges and proposes solutions for omics integration in an effort to raise support and awareness of omics integration within the scientific community. It is hoped that this report will initiate new collaborative efforts that harness the vast amount of knowledge embedded in disparate data sets and promote training of more multidisciplinary scientists better positioned in the science of omics integration (integromics).

## Workshop overview

To identify key limiting factors and challenges in integromics and provide actionable solutions to overcome such roadblocks in the context of biology and diseases, the workshop was structured to ground discussions upon three case studies - personal omics profiling [11], multi-omics pathway analysis of cardiovascular-specific circadian clock [12], and glycoproteomics [13]. In addition, experts from the Framingham Heart Study presented a “lessons learned” talk on identifying risk factors for heart disease and its associated studies using omics-based technologies on a much larger patient population [14, 15]. Next, workshop participants broke off into multi-disciplinary groups for further discussion in order to develop integrative solutions to address three major areas of challenges (clinical, informatics, and technology) identified. For example, questions were raised by the participants during rounds of discussions, including: (1) Can omics improve the odds ratio for diabetes or heart disease prediction in cardiovascular research? (2) Can omics science provide the context for cancers that begins as genetic aberrations? Collectively, six major recommendations for facilitating omics integration were put forth and summarized below.

## Case studies

### Personal omics profiling (case study 1)

The case study described by Dr. Michael Snyder from Stanford University illustrated how integration of different omics data can facilitate a shift from disease treatment to prevention based on his own experience. Discussed was how longitudinal personalized omics profiling (POP) from analysis of the genome, epigenome, transcriptome, proteome and metabolome (“Snyderome”) can collectively provide useful information that otherwise could not be gleaned from any single individual omics domain (data sets) alone. The “Snyderome” included routine measurements interspersed with dense sampling during states of infection. Integrative analyses of the data revealed an increased insulin biosynthetic pathway that spiked during states of viral infections [11]. The data further indicated Dr. Michael Snyder to be at an increased risk of type 2 diabetes, despite having no known family history of the disease, which subsequently proved true. This highlights the fact that following multiple omics components longitudinally may provide valuable information about disease risk, drug sensitivity, and other components of personalized medicine.

This POP study simultaneously illustrated the potential of omics integration. Clearly, methods exist to shift less studied areas of medicine from hearsay and conjecture to data-established-truth. Yet, POP studies are hardly scalable across a population due to an analysis cost of $10,000 per sample. Furthermore, progress in POP research requires people to allow the collection of their omics profiles. This is a delicate subject as the collection of so much data will increase the likelihood of false positives and induce undue or premature emotional strain. The so-called, “democratization of data”, namely the shift from expert protectionism to people governing their own data, has led to the possibility of better decision-making which might significantly impact the choices they make day-to-day. Although this can be done in medicine, the challenge remains to protect human subjects without hindering research, while restraining clinical adoption until clear data-driven-truths have been clinically validated.

### Pathways and targets to modulate clocks (case study 2)

Dr. John Hogenesch from University of Pennsylvania discussed the utility of omics integration to identify clock-modifying genes and pathways. The circadian clock regulates many aspects of biology, including core body temperature, organ function, heart rate, and blood pressure, among others. Clocks are present in most of the body’s cells and interestingly most cancers appear to have lost their circadian clocks.

Omics approaches that include whole-genome siRNA circadian genomic screens, gene expression data, and protein-protein interaction data are used to identify clock-modifying genes and define their mechanistic and functional attributes [16]. The insulin signaling pathway is one of the most significant clock-modifying pathways identified by such an approach. Dr. John Hogenesch discussed the use of Bayesian integration strategies to help assess whether the evidence provided by a given result indicates that the gene is a core clock component. Additional discussion on major challenges for integrating omics results include the use of different synonyms by the scientific community (e.g., multiple names for a given gene and/or its variants, and access to high-quality standardized data sets for "trustworthy" analyses).

### Glycoproteomics (case study 3)

Drs. Gerald Hart and Jennifer Van Eyk from Johns Hopkins University discussed the fields of glycobiology, highlighting the critical nature of integrative approaches since one omics domain cannot adequately explain the underlying biology. Dr. Gerald Hart estimated that 90% of proteins are glycosylated, and glycosylation is involved in nearly all cellular activities and metabolic processes. He also noted that post-translational modifications (PTMs), such as glycosylation, greatly expand the genetic code’s chemical diversity, and hence, function cannot be inferred through genomics approaches alone. “Glycomics” is defined as the study to characterize or quantify the glycome of a cell, tissue, or organ. Glycome complexity is a reflection of cellular complexity and the collective tools of genomics, proteomics, lipidomics and metabolomics are required for functional characterization. Challenges to the integration of glycomics include a lack of integration of glycan data into mainstream databases, a lack of standardization across existing glycomic databases, and a lack of clarity regarding different levels of glycan “structure” in published literature. A further challenge is the paucity of measurement tools for site-specific identification and quantitation of glycoproteomics.

### The Framingham heart study (lessons learned)

The Framingham Heart Study was initiated in Framingham, Massachusetts in 1948 to understand the underlying causes of cardiovascular disease (CVD). The study aimed to investigate the expression of coronary disease in a normal population, determine factors that predispose individuals to develop CVD, and evaluate new screening tests (e.g., electrocardiography, blood metabolites). Currently, the Framingham Study incorporates a systems biology approach to biomarker research [i.e., CVD Systems Approach to Biomarker Research (SABRe) initiative], aiming to identify biomarker signatures of CVD and its major risk factors using omics technologies. Dr. Andrew Johnson from NHLBI summarized omics data collected to date, in which studies have profiled three generations of families across thousands of phenotypes with many of them being longitudinal. Specific data collected include 8,500 genome-wide association studies, 7,000 cell line analyses, 300 whole exome sequences, 1,000 whole-genome sequences, 5,000 DNA microarrays, 2,000 metabolomics analyses, and ongoing data collection with induced pluripotent stem cells, DNA methylation, computed tomography scans, and magnetic resonance imaging. Challenges identified in the Framingham Heart Study include data acquisition (e.g., throughput, cost, and sample tracking/batch effects), storage (e.g., results, storage demands, raw data in one place for cross-comparison, etc.), and limitations with data processors, competing needs on servers, costly renewal of outdated resources, and security issues.

### Roadblocks in integrating omics knowledge in biology and medicine

Discussions regarding roadblocks and challenges in omics science that took place following the presented case studies are outlined below with a focus on three main areas - clinical utility, informatics, and technology.

### Clinical utility challenges

Two fundamental challenges that were identified for the integration of omics into medicine included (1) disseminating, managing, and interpreting omics data in a clinical context, and (2) ensuring that omics results have added value to existing paradigms of patient care. Providing a solution to these problems should allow for enhanced preventative, diagnostic, and prognostic procedures [17]. The democratization of multi-omics data is a key aspect of the integration of omics data in medicine. While the physical barriers to access, management, and transfer of data have been removed through the digitalization of data files, clinical utility of research data is limited by privacy and other barriers, justly placed to prohibit the abuse of protected health information. However, the ease of disseminating, managing, and interpreting massive amounts of omics data would allow for quicker application of integrative omics knowledge to clinical practice.

Transforming and incorporating data derived from different omics approaches into a defined clinical context is essential, but remains complex and problematic [18, 19]. Genomic scans, for example, have started to identify more and rarer variants in addition to common SNP variants [20], and when different commercial platforms are used to molecularly analyze a common sample, variability is often found in their risk prediction capacities [21]. This variability most likely lies in data interpretation models that incorporate different assumptions during data processing and widespread problems of overfitting high dimensional data with an extremely large number of molecular measurements relative to limited sample size [19]. This begs the question of how well a genetic variant correlates to a specific disease condition and whether predicted disease risks have any clinical validity. In the age of declining genotyping costs and retail genome sequencing kit, consumers can now obtain data on their own personal DNA, and patient expectations of clinicians providing useful genetic information are soaring. Therefore, a disconnect is growing between the realistic, operable utilities of omics sciences and the expectations of patients with little clarity on how to bridge the gap. Finally, legitimate concerns about how to keep data and results private and secure are becoming more prominent.

The second major clinical challenge lies in determining, through appropriate studies, whether the new omics findings add incremental value to current clinical practices or clinical decision making. While multiple omics technologies can potentially discover a host of biological candidates from samples, their clinical utility requires rigorous validation. Hence, discovery-based omics research should seek to maximize the signal-to-noise ratio of a biomarker candidate(s) in order to produce fewer false leads [19]. Furthermore, it is important to distinguish the causes of pathogenesis versus markers that indicate disease phenotypes, since causes are often treatable and have robust associations (e.g., LDL and atherosclerosis [22]), whereas markers of disease are the often most powerful predictors. Although the markers of diseases can guide diagnosis and treatment, their effects are not a direct target for treatment (e.g., you can treat LDL, but you do not treat Troponin). Cholesterol was studied for over 100 years prior to becoming a clinically useful biomarker. However, it is uncertain that any new biomarker candidates from omics studies alone or in combination to cholesterol perform better than cholesterol alone. Such complex barriers need to be adequately addressed to be of help in actionable clinical decision-making.

### Informatics challenges

Three major challenges identified in informatics that limit the integration of omics data in the clinic were (1) the development of more mature models of cellular processes that incorporate non-commensurate omics data types [23, 24], (2) data storage limitations and organization of fragmented data sets, and (3) a shortage of multidisciplinary scientists with training in biology, computer science, informatics and statistics.

Omics integration includes the incorporation of multiple omics data types into a comprehensive model that accurately describes biological processes. The simplest model assumes the “central dogma” and maps transcripts and proteins to gene sequences. Slightly more sophisticated models entail quantitative information and use correlations across molecular entities. As each “ome” reflects a distinct biological domain (e.g., transcripts, proteins, metabolites), the resulting datasets represent the measurements of various underlying variables on different scales. For example, transcriptional and translational profiles for mRNA transcripts and corresponding proteins are often but not always the same [25–27]. To capture both the temporal and spatial dynamics of biomolecules embedded within complex biological relationships, the most complex models must appropriately integrate all pertinent, distinct measurements of the various Omes. However, the modeling of non-commensurate data types comprised of nonlinear relationships and multivariate signals is extremely complex, and current computational algorithms and statistical procedures are limited in this capacity. Additionally, the non-synonymous naming systems for the myriad of biological molecules in the various Omes further complicate algorithm development and inhibit omics integration. As discussed previously, modeling would be greatly aided by the standardization of gene names (e.g., circadian clock genes). Once a model is established, faster and more efficient methods are required to validate computational results in cellular and animal model systems, representing a huge challenge in the field of integrative omics science [28].

This specific challenge is particularly difficult to address, involving many aspects of the scientific and clinical disciplines dependent on the diseases, including but not limited to:

- relative risk of disease or adverse outcome is often arbitrarily assigned,
- association does not necessarily equal prediction,
- insufficient sample numbers in some studies,
- difficult to extrapolate from n = 1 to a population and to model the environment, and
- modeling needs to be performed by computers and not by physicians, with results translated to a scale that physicians can easily understand (e.g., 10-year coronary heart disease risk).

The second bioinformatics challenge for omics integration involves the storage of large, heterogeneous datasets generated from multiple high-throughput omics platforms. With the continued development of more sophisticated instrumentation for data acquisition, the amount of data generated is exponentially rising, along with the demand for data storage. As the usage of stored data occurs at distinct levels (e.g., raw data vs. mass spectrometry search results files in proteomics, or raw nucleotide sequence reads vs. variant calls in vcf format in genomics) specific to a particular expertise in the multi-disciplinary end user pool (e.g., computer scientists vs. genome biologists), data storage infrastructure should be stratified and specifically tailored to meet the needs of end users. If storing all data is cost-prohibitive, the difficulty lies in determining which data are the most valuable to keep. Furthermore, datasets are heterogeneous with respect to both intra-omics (e.g., proteomic datasets from different file formats) and inter-omics (e.g., genomic vs. proteomic datasets) acquisition protocols. This results in a storage infrastructure that is fragmented and disjointed, thereby hindering cross-comparison and retrograde use by the scientific community. Security and privacy of stored clinical data is an additional issue for avoiding ethical concerns.

The participants collectively put forth recommendations to overcome informatics barriers by:

- establishing data standards for all types of omics data files (e.g., cite genomics and proteomics papers),
- changing access to data [29] to protect research subjects without hindering valuable research opportunities,
- completing the incomplete reference databases (~1/3 of SNPs in dbSNP), such as using proteomics data to confirm/verify gene annotation [30], and adding PTMs that are not routinely integrated in mainstream databases,
- calculating some key parameters for data processing and storage, such as how many times will a raw file be processed? How long will it need to be stored? How frequently do data analysis methods change?
- providing sufficient incentive to data generators for data deposition into publicly accessible repositories although great stride has been made in the past few years such as dbGAP and ProteomeXchange [31], and
- overcoming data storage and computing power limitations.

The third major bioinformatics challenge is primarily driven by technology. Rapidly evolving analytical methods unleash new measurements which in turn give rise to new types of data and data analysis. Hence, there is a constant requirement for scientists including bioinformaticians to keep up with the developing technologies and methodologies. Most experts in the field have experience in a single omics technology, such as calling mutations in next-generation sequencing data or extracting peptides from mass spectra, and those who specialize in the next higher level of data integration are rare. A combination of reasons contribute to this dearth including: rapidly changing technologies that keep bioinformaticians from continually specializing in the analysis of one molecular moiety, insufficient biomedical informatics training opportunities, and the transient nature of the interface between technology development and disease-specific research. Major adjustments to the vision and expanding the training of medical bioinformatics research community are highly recommended and required to surpass these obstacles, even though informatics training opportunities related to NIH’s BD2K initiative and others have been added more recently to address this challenge.

### Technological challenges

Two major technological challenges that were recognized to limit omics integration into medicine were (1) a lack of reproducibility of data acquired through non-uniformly standardized sample preparation, including a lack of understanding of the impact of pre-analytical variables on samples [32], and inconsistent instrument performance [19], and (2) a lack of high-throughput and multiplexing methods that make parallel measurements of multiple types of analytes for handling large clinical studies. Addressing such obstacles, the scientific community has come a long way to demonstrate the analytical robustness of genomic, proteomic, and metabolomic workflows, including data analysis pipelines as witnessed by a flurry of standardization/harmonization activities during the last two decades in several omics areas including Genomic Standards Consortium, CPTAC, HUPO and ABRF [33–40]. Furthermore, there have been significant technological advances in measuring genomic variants, proteins and peptides, and small molecule metabolites that include next-generation genomic sequencing, immuno-multiple reaction monitoring mass spectrometry, flow cytometry, and protein microarrays [41–44]. There is no doubt that technologies will continue to be improved/developed to increase sensitivity, specificity and throughput, making it feasible to measure every molecule at the single cell level. To apply multiplexing and high throughput methods in clinical studies, researchers need to ensure that the appropriate technologies/platforms and bioinformatic analyses are analytically robust and standardized, and can be validated in an independent lab and/or in a separate set of clinical samples.

## Recommendations for successful omics integration

Following rounds of discussions, six major recommendations for facilitating omics integration to address the identified roadblocks described above were put forth by workshop participants and summarized below.

1. Committed funding for the education of multi-disciplinary teams is needed. Clinicians, clinical scientists, basic scientists, and bioinformaticians need to be educated in these disciplines, and form collaborative, multi-disciplinary teams to carry out omics integration from discovery to the patient. Omics sciences are inherently integrative of multiple specialties. Therefore, all phases of discovery efforts, including sample procurement, experimental design and bio-interpretation, and all phases of clinical translation including clinical trials and implementation into clinical procedures must be performed by a multi-disciplinary team of investigators. From this, appropriate epidemiological and statistical measures should be applied for determining whether a newly discovered marker or panel of markers adds value to pre-existing clinical regimes of risk prediction, diagnosis and prognosis. Furthermore, end users need to be educated on the realistic utilities of omics results at each stage of omics development. This can be accomplished via public seminars or via genetic counselors acting as a liaison between clinicians and patients. This will lessen unrealistic expectations of the public for physicians to infer patient risk from the results of omics studies. In the long term, committed funding to create a new discipline of omics sciences is needed, providing rigorous training in the omics sciences in order to create a group of specialized experts to propel the field forward. Fellowships are needed for young scientists in the field of omics sciences to train future experts. Specifically, there is a need for the development of informatics training centers that produce experts who derive meaning from large omics datasets, including data curators and wranglers.
2. Committed and sustained funding for technology development is needed. In particular, further developments are needed in mass spectrometry instruments and technologies (e.g., top-down MS) in order to sequence deeper proteomes and/or metabolomes, and to allow for high throughput multiplexed analysis.
3. Sample preparatory procedures and acquisition must be standardized to allow for reliable cross-comparison, sharing and integration of large omics datasets and for whole-omics profiling from the same sample.
4. The development of an unifying resource is needed to permanently store data in a coordinated and structured manner. This resource would provide security, privacy and consensus on how data are stored and accessed by the community. This is critical for the integration of omics sciences and one where the National Institutes of Health (NIH) can play a significant role.
5. Mature models for integrating non-commensurate data types are needed. Algorithms must be developed for data compression, integration, querying and display to handle the distinct data types of omics sciences. Quality control algorithms should be developed for data format and exchange, and natural language data mining.
6. A consensus needs to be developed in order to create validity and value for integrating omics findings into clinical guidelines. Useful, reliable and valid metrics for establishing association and prediction in diagnostic and prognostic studies need to be utilized. Moreover, calculations for diagnostic and prognostic purposes need to be locked down and automated within a laboratory in order to remove any inconsistencies stemming by physician bias or interpretation. Translating scores to a scale that physicians can understand and converting to a single scale that can be modified over time is very important in this process [19, 45].

## Conclusion

Omics science has transformed biology and has the potential of transforming medicine. This workshop was a first step on opening a dialogue amongst scientists and clinicians in relevant omics disciplines to (1) update recent progress and further emphasize the importance of omics science and its potential in transforming biology and future clinical practice, (2) discuss barriers in omics integration existent in a variety of forms, and (3) put forth recommendations to overcome such barriers to enable the science to move forward.
